# Heterogenous circulating miRNA changes in ME/CFS converge on a unified cluster of target genes: A computational analysis

**DOI:** 10.1371/journal.pone.0296060

**Published:** 2023-12-29

**Authors:** Mateusz Piotr Kaczmarek

**Affiliations:** Independent researcher, Toruń, Poland; University of Nebraska-Lincoln, UNITED STATES

## Abstract

Myalgic Encephalomyelitis / Chronic Fatigue Syndrome is a debilitating, multisystem disease of unknown mechanism, with a currently ongoing search for its endocrine mediators. Circulating microRNAs (miRNA) are a promising candidate for such a mediator and have been reported as significantly different in the patient population versus healthy controls by multiple studies. None of these studies, however, agree with each other on which specific miRNA are under- or over-expressed. This discrepancy is the subject of the computational study presented here, in which a deep dive into the predicted gene targets and their functional interactions is conducted, revealing that the aberrant circulating miRNAs in ME/CFS, although different between patients, seem to mainly target the same specific set of genes (p ≈ 0.0018), which are very functionally related to each other (p ≲ 0.0001). Further analysis of these functional relations, based on directional pathway information, points to impairments in exercise hyperemia, angiogenic adaptations to hypoxia, antioxidant defenses, and TGF-β signaling, as well as a shift towards mitochondrial fission, corroborating and explaining previous direct observations in ME/CFS. Many transcription factors and epigenetic modulators are implicated as well, with currently uncertain downstream combinatory effects. As the results show significant similarity to previous research on latent herpesvirus involvement in ME/CFS, the possibility of a herpesvirus origin of these miRNA changes is also explored through further computational analysis and literature review, showing that 8 out of the 10 most central miRNAs analyzed are known to be upregulated by various herpesviruses. In total, the results establish an appreciable and possibly central role for circulating microRNAs in ME/CFS etiology that merits further experimental research.

## Introduction

Myalgic Encephalomyelitis / Chronic Fatigue Syndrome (ME/CFS) is an acquired, often post-viral, chronic, multisystem disease affecting approximately 1% of the population [1]. It causes severe, lifelong disability and is characterized by exceedingly low quality of life [2, 3]. No approved treatments are currently available, and its etiology is poorly understood. The importance of increased ME/CFS research has recently been underlined by the emergence of “Long COVID”, another debilitating, untreatable, post-viral disease bearing a striking resemblance to ME/CFS, with some arguing for the unification of the two as one disease [4–6].

The systemic nature of the illness as well as direct experimental results using ME/CFS plasma, strongly imply the involvement of endocrine factors in the disease mechanism [7, 8]. However, the search to identify said endocrine factors is still ongoing.

MicroRNA (miRNA) are short, non-coding RNA capable of modulating a cell’s gene expression, mainly via binding to mature messenger RNAs and interfering with their translation or tagging them for degradation [9]. In recent years, circulating extracellular miRNAs, carried by exosomes or otherwise, were found to constitute an endogenous, highly regulated and complex form of cell-to-cell communication and interregulation, both in a paracrine and endocrine manner [10].

Thus, altered circulating miRNA composition and levels are a promising candidate for a complete or partial endocrine mediator of the ME/CFS disease mechanism, a notion bolstered by the observed elevated levels of circulating exosomes in patients’ plasma [11, 12].

However, as will be shown below, experimental studies measuring circulating miRNAs from ME/CFS patients have produced strongly diverging results, failing to establish a consensus circulating miRNA signature of the disease. This might imply the unimportance of circulating miRNAs to ME/CFS or be a reflection of a wide disease heterogeneity. However, another possibility is the notion that seemingly divergent miRNA combinations of each patient might target similar sets of genes or impact the same pathways, though through different regulatory intermediaries. To explore this option, the present study is conducted, aggregating existing data of circulating miRNA levels in ME/CFS and re-analyzing them in new, in-depth computational ways.

## Methods

**Fig 1:**
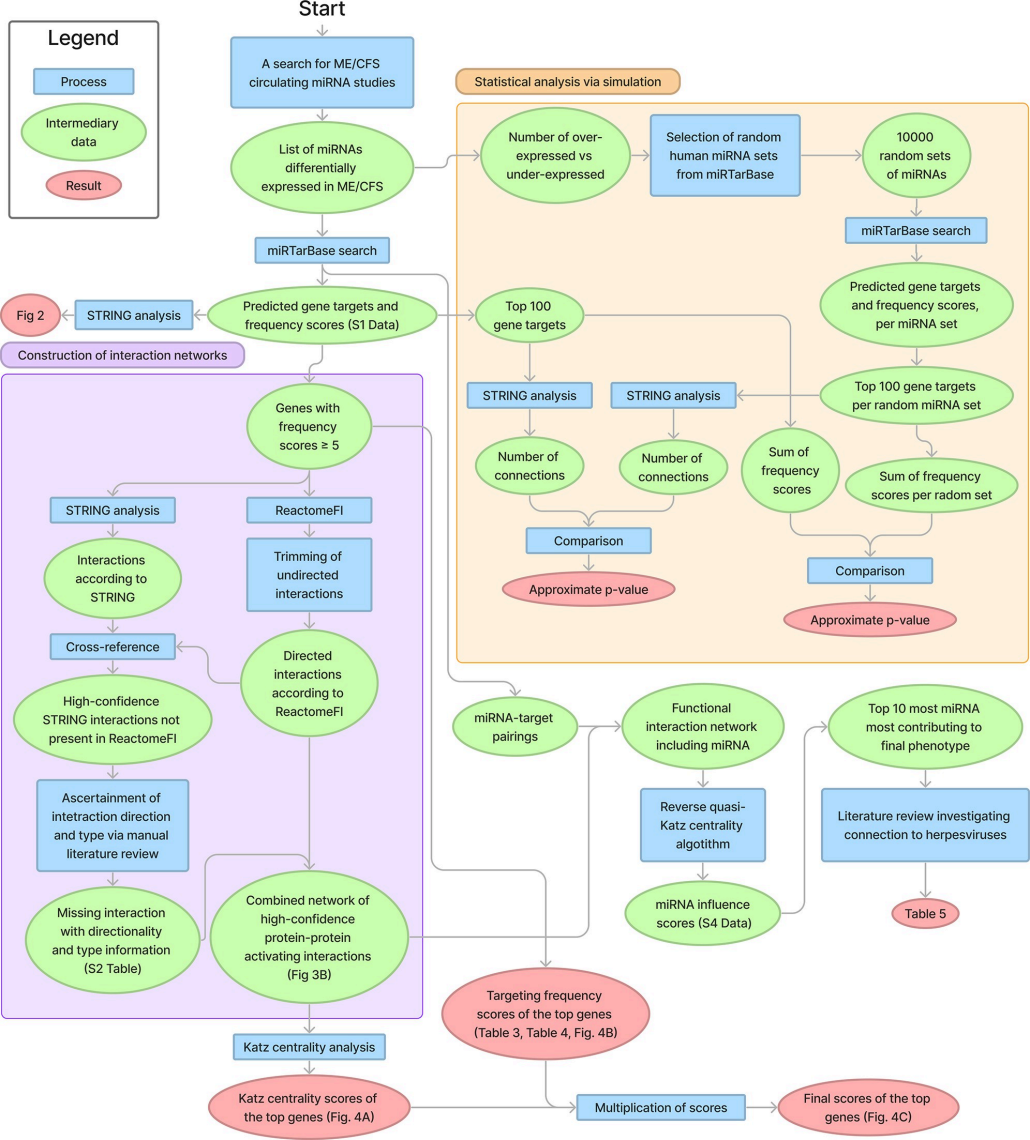
Methodology flowchart.

The full methodology including the flow of data has been illustrated in Fig 1, and was carried out as follows.

### Gathering miRNA data from primary literature

A literature search for “Myalgic Encephalomyelitis miRNA” and “Chronic Fatigue Syndrome miRNA” was performed, and subsequently narrowed down to only studies reporting circulating microRNA measurements.

All miRNAs reported to be differentially expressed between ME/CFS and healthy controls were pooled together, regardless of their fold-change, p-value and circumstances under which they were measured (at rest or after exercise, in one study), noting only which were overexpressed and which underexpressed.

### miRNA target prediction

The resulting list was used as input for searching miRTarBase, a manually curated database of experimentally-verified miRNA-target interactions [13]. Specifically, used was the 2020 edition of miRTarBase, the MTI database file representing all recorded interactions.

For each human gene it was counted how many miRNAs from the ME/CFS list targeted it. Overexpressed (high) and underexpressed (low) miRNA targeting occurrences were counted separately, and the difference between those two counts constituted the final miRTarBase targeting frequency score for each gene.

### Analyzing target protein functional interconnectivity

Initial analysis of the functional relationships between the resulting genes and visualization thereof was performed using the STRING database of protein-protein interactions [14].

Statistical analysis was performed via simulation, using 10000 random miRNA sets. The sets were all of the same number of high and low miRNAs as the ME/CFS set, picked from miRTarBase at random, using quantum true randomness via ANU QRNG [15]. The resulting 10000 sets were checked against miRTarBase and target genes scored in the exact same fashion as the ME/CFS set. For each set, the summed score of the top 100 genes as well as the number of connections between them via STRING analysis were counted and compared to the ME/CFS set to calculate approximate p-values.

### Finding most downstream target proteins by using directional pathway information

For functional pathway analysis of the implicated genes from the ME/CFS set, first a subset of most impacted genes (frequency score ≥ 5) was selected. Next, functional interactions were drawn between them using ReactomeFI in Cytoscape [16, 17]. The resulting network was trimmed of undirected interactions, and bidirectional ones were split into discrete edges. The resulting directed graph was cross-referenced with the undirected graph for the same set of genes produced by STRING using threshold = 0.95, and the missing connections were investigated via manual literature review (constrained to human cell studies only) to ascertain their directionality and type (activation/inhibition). To further prepare the network for centrality analysis, inhibiting edges were removed, along with any edges of uncertain type, resulting in a trimmed network of high-confidence activating interactions only. Said network was then analyzed using the Katz centrality algorithm with coefficients α = 0.1 and β = 1. For each gene its centrality score was multiplied with its initial miRNA targeting frequency score, to yield the final combined score, reflecting how much it is impacted both directly and indirectly.

### Finding miRNAs most heavily contributing to the final phenotype

Specific influence of each upregulated miRNA over the final phenotype was investigated by using the aforementioned directional graph in reverse, in a simple algorithm similar to Katz centrality as follows:

$$I_{miRNA}=\sum_{g=1}^{n}(s_{g}\times \sum_{p=1}^{m}\alpha^{l_{p}})$$

Where:

I_miRNA_—influence score of a given miRNA

n—number of genes the miRNA influences directly or indirectly

s_g_—previously established final score of the given gene

m—number of unique paths between the miRNA and gene

l_p_—length of each path

α - attenuation factor (equal to 0.1 for this study)

In other words, for each miRNA-gene pair, all unique paths between them in the graph were evaluated, and a constant attenuation factor raised to the power of their length (just like in Katz centrality). The sum of these, reflecting how much a given miRNA impacted a given gene, was then multiplied by the gene’s final score (reflecting its importance in the final phenotype). The sum of all of these, for all genes, constituted the final influence score of the given miRNA.

## Results and discussion

Four studies meeting the criteria were identified, reporting a total of 56 circulating microRNAs differentially expressed between ME/CFS patients and healthy controls [18–21]. 37 of those were elevated in ME/CFS and 19 were decreased. Most of the miRNAs were unique to each study, with only 4 being reported by two separate publications. No miRNAs were reported by three or more studies. No miRNAs were reported high in one study and low in another (Table 1). Demographics of the subject from these studies are presented in Table 2.

**Table 1:** miRNAs differentially expressed between ME/CFS patients and healthy controls.

Elevated miRNAs	hsa-let-7d-5p, hsa-let-7g-5p, hsa-miR-15a-5p, **hsa-miR-21-5p**, hsa-miR-29a-3p, hsa-miR-34a-5p, hsa-miR-92a-3p, hsa-miR-93-5p, **hsa-miR-126-3p**, **hsa-miR-127-3p**, hsa-miR-130a-3p, hsa-miR-136-3p, hsa-miR-140-5p, hsa-miR-142-5p, hsa-miR-143-3p, **hsa-miR-150-5p**, hsa-miR-181b-5p, hsa-miR-185-5p, hsa-miR-200c-3p, hsa-miR-223-3p, hsa-miR-320e, hsa-miR-331-3p, hsa-miR-370-3p, hsa-miR-374a-5p, hsa-miR-374b-5p, hsa-miR-381-3p, hsa-miR-423-5p, hsa-miR-493-5p, hsa-miR-548ax, hsa-miR-548j-5p, hsa-miR-4454, hsa-miR-4532, hsa-miR-5581-5p, hsa-miR-6076, hsa-miR-6717-5p, hsa-miR-6875-5p, hsa-miR-7975
Decreased miRNAs	hsa-let-7g-3p, hsa-miR-16-2-3p, hsa-miR-26a-1-3p, hsa-miR-33a-5p, hsa-miR-126-5p, hsa-miR-183-5p, hsa-miR-203a-5p, hsa-miR-369-3p, hsa-miR-450b-5p, hsa-miR-486-5p, hsa-miR-607, hsa-miR-641, hsa-miR-3065-3p, hsa-miR-3620-3p, hsa-miR-4433a-5p, hsa-miR-5187-3p, hsa-miR-6507-3p, hsa-miR-6800-3p, hsa-miR-6819-3p

**Bold**—independently reported by two distinct studies. All others are unique to their respective source study.

**Table 2:** Patient demographics of the identified miRNA studies.

study	sample size	age	gender	disease severity	inclusion criteria	exclusion criteria
Brenu et al, 2014 [18]	20	44.5±6.0 years	no info	no info	1994 “Fukuda” [22]	smokers, pregnant/breast-feeding, immobile, having autoimmune, thyroid or cardiac related disorders
Almenar-Pérez et al, 2020 [19]	15	38 to 53 years, avg 46.8	female only	severe	1994 “Fukuda” [22] and/or CCC [23]	UK ME/CFS biobank exclusion criteria*
Nepotchatykh et al, 2020 [20]	43	58 ± 2.3 1st cohort, 49.2 ± 2.1 2nd cohort	16 male, 27 female	all	CCC [23]	no info
Blauensteiner et al, 2021 [21]	58	19 to 58 years	14 male, 44 female	all	1994 “Fukuda” [22] and/or CCC [23]	UK ME/CFS biobank exclusion criteria*

*UK ME/CFS biobank exclusion criteria:

taken antiviral medication or drugs known to alter immune function in the preceding 3 months; had any vaccinations in the preceding 3 months; had a history of acute and chronic infectious diseases such as hepatitis B and C, tuberculosis, HIV (but not herpes virus or other retrovirus infection); another chronic disease such as cancer, coronary heart disease, or uncontrolled diabetes; a severe mood disorder; been pregnant or breastfeeding in the preceding 12 months; morbidly obese (BMI ≥ 40)

### Diverse miRNA signals converge on a set of highly functionally interconnected genes

**Fig 2:**
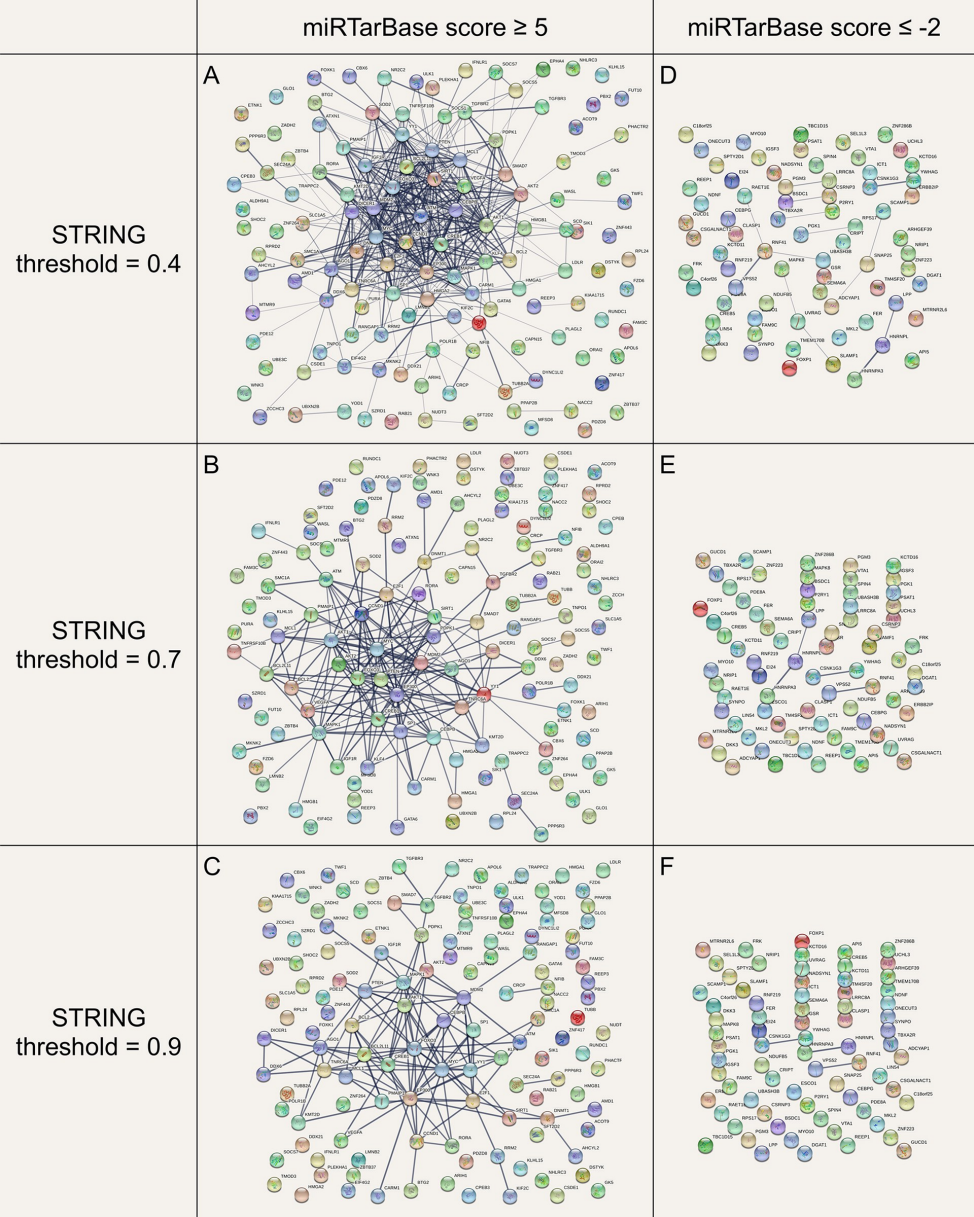
Functional interactions between implicated genes via STRING analysis. **(A-C)** Genes most targeted by elevated miRNA. **(D-F)** Genes most targeted by downregulated miRNA.

Following miRTarBase analysis, of the genes targeted by elevated miRNAs, 128 had a score ≥ 5 (Table 3), and formed a large cluster of functional interconnectivity, as revealed by STRING analysis (Fig 2A–2C). This cluster contains all of the most highly targeted genes, and holds up even when restricting STRING to very high confidence interactions only (threshold = 0.9), thus supporting the idea of circulating miRNAs eliciting similar cellular changes in ME/CFS, despite the specific miRNA signature differing considerably from patient to patient. Conversely, the genes targeted by downregulated miRNAs did not have highly negative scores (Table 4), and showed virtually no clustering via STRING, even with a fairly low threshold of 0.4 (Fig 2D–2F). This suggests (though not definitively demonstrates) that they are unlikely to play a central role in the pathology of the disease.

**Table 3:** Genes most targeted by elevated circulating miRNA.

Total miRTarBase frequency score	Genes
11	VEGFA
10	-
9	CCND1
8	ZNF264, SHOC2
7	BCL2, SIRT1, MKNK2, E2F1, KMT2D, AGO1, GK5, TGFBR2, HMGA2, YOD1
6	ZBTB37, RPRD2, TUBB, IFNLR1, IGF1R, MCL1, PLAGL2, SOD2, HMGB1, WASL, KLHL15, SMC1A, MYC, ACOT9, APOL6, EPHA4, PDPK1, SCD, MFSD8, GATA6, DICER1, EP300, NFIB, FZD6, TNFRSF10B
5	AKT2, ZADH2, TNRC6A, SIK1, SEC24A, CARM1, ATXN1, LMNB2, SP1, PLEKHA1, SLC1A5, PMAIP1, ALDH9A1, RORA, KLF4, EIF4G2, PPP6R3, RPL24, ARIH1, MTMR9, TUBB2A, DDX6, TMOD3, UBE3C, SOCS7, CBX6, CPEB3, NR2C2, ZCCHC3, ZNF417, ATM, PBX2, TGFBR3, PLPP3, UBXN2B, ETNK1, GLO1, KIF2C, BTG2, FUT10, FOXO3, PHACTR2, ULK1, RANGAP1, RUNDC1, SZRD1, DYNC1LI2, AHCYL2, NACC2, YY1, REEP3, AKT1, WNK3, RAB21, ZNF443, FAM3C, CSDE1, DDX21, LDLR, SOCS1, LNPK, MAPK1, AMD1, ORAI2, CEBPB, PDZD8, ZBTB4, NHLRC3, PURA, RRM2, CREB1, PTEN, DNMT1, DSTYK, TNPO1, BCL2L11, POLR1B, CAPN15, CRCP, MDM2, SOCS5, SMAD7, SFT2D2, NUDT3, TWF1, FOXK1, PDE12, TRAPPC2, HMGA1

**Table 4:** Genes most targeted by downregulated circulating miRNA.

Total miRTarBase frequency score	Genes
-2	ONECUT3, TBXA2R, C4orf26, UVRAG, NDUFB5, SNAP25, NDNF, FRK, NADSYN1, MRPL58, CSRNP3, CEBPG, ESCO1, BSDC1, ZNF223, VPS52, LRRC8A, CSGALNACT1, KCTD16, P2RY1, PGK1, ADCYAP1, GUCD1, REEP1, GSR, SYNPO, FER, FAM9C, HNRNPL, RNF219, PGM3, PSAT1, TM4SF20, RAET1E, VTA1, ARHGEF39, NRIP1, UBASH3B, UCHL3, YWHAG, DKK3, RPS17, RNF41, SCAMP1, ERBIN, MAPK8, PDE8A, KCTD11, API5, SLAMF1, LIN54, TMEM170B, C18orf25, CREB5, MTRNR2L6, SEL1L3, TBC1D15, SEMA6A, HNRNPA3, ZNF286B, SPTY2D1, IGSF3
-3	EI24, CSNK1G3, MYO10, CRIPT, LPP, MKL2, FOXP1, DGAT1, CLASP1
-4	SPIN4

Full list of all genes and their scores is available in S1 Data.

To further investigate the statistical significance of the notion that diverse miRNA signatures converge on a singular cluster of functionally interconnected genes, a simulation was performed using 10000 random sets of 37 high and 19 low miRNAs. They were compared with the ME/CFS set in two metrics. First, the total sum of scores of the top 100 genes identified by miRTarBase analysis, indicating convergence on a specific set of genes. And second, the number of connections between those genes via STRING, reflecting their functional interconnectedness. In the sum test, only 18 controls scored higher than the ME/CFS set, resulting in a p-value ≈ 0.0018. For the STRING connection number test, 0 random controls had more STRING connections than the ME/CFS set, regardless of the STRING threshold being set to 0.4, 0.7 or 0.9. This suggests a p-value ≲ 0.0001. Full simulation data are available in the data repository. Altogether the results of this simulation demonstrate a remarkably statistically robust functional clustering of the targets of miRNAs overexpressed in ME/CFS, highly unlikely to arise by chance, pointing to functional convergence at the pathway level.

### Directional topological analysis of the functional connections between implicated genes converges on similar targets as miRNA targeting frequency ranking

**Fig 3:**
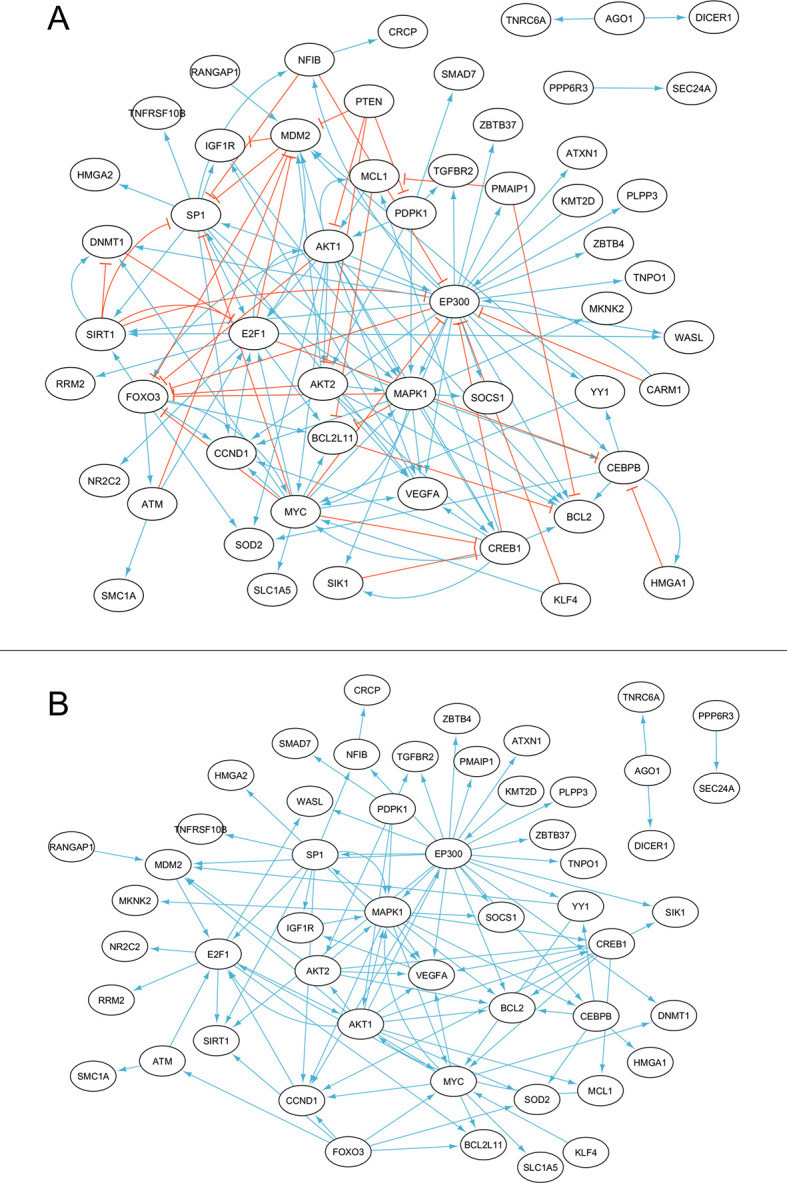
Directional protein-protein interaction networks. **(A)** Both activating and inhibiting edges present. **(B)** Activating edges only; used for Katz centrality analysis.

**Fig 4:**
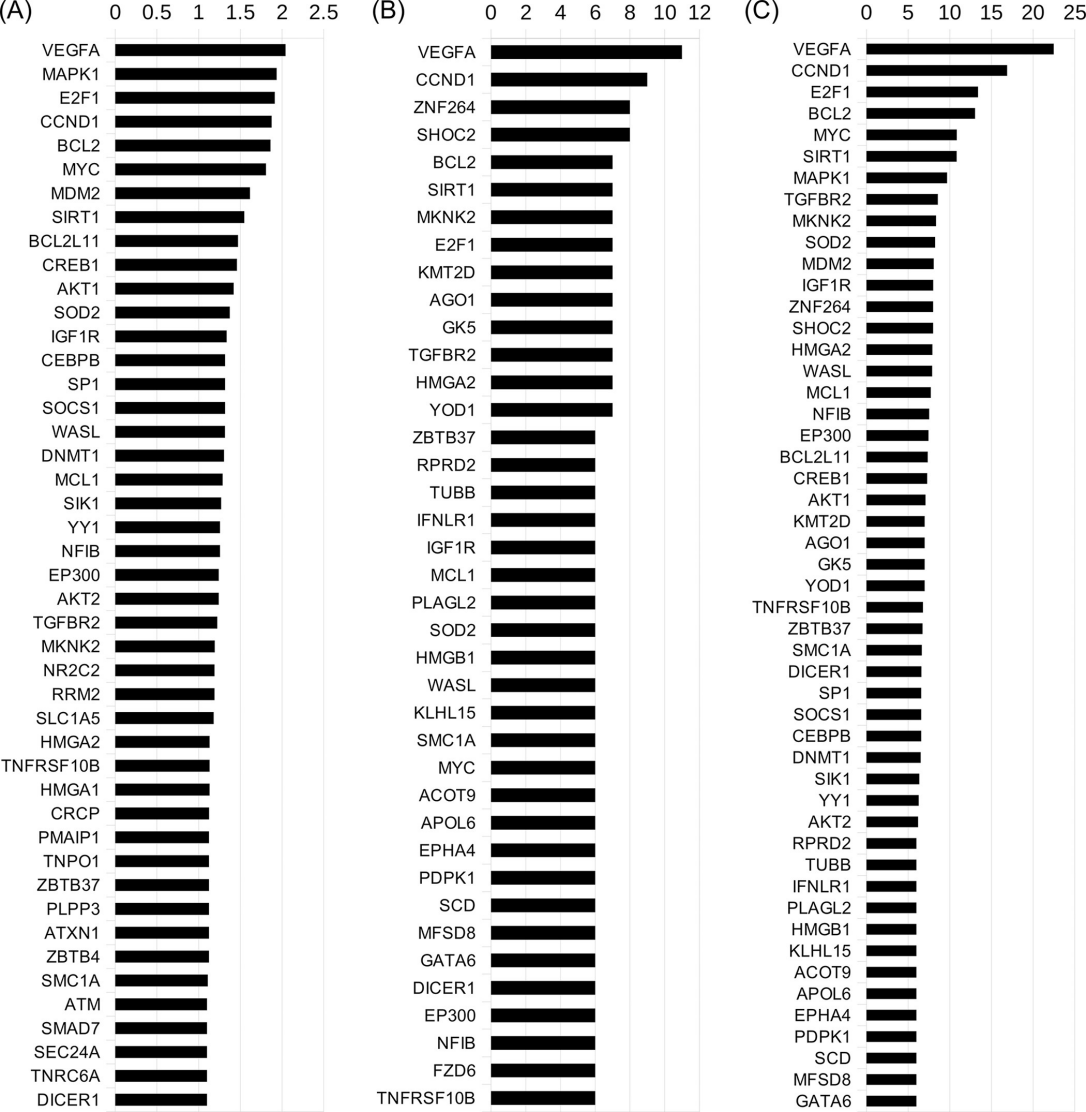
Ranking of topmost inhibited genes. **(A)** Genes ranked by their Katz centrality score (scores > 1 pictured). **(B)** Genes ranked by miRTarBase frequency score (for comparison; same as Table 3; only scores ≥ 6 pictured). **(C)** Top 50 genes with the highest final score (miRTarBase score multiplied by Katz centrality score). Full list available in S2 Data.

To ascertain which genes lie most downstream in this complex network of interactions, a new interaction network, including directionality information as well as activation/inhibition type (Fig 3A), was constructed by combining protein-protein interaction data from multiple databases and manual literature review (S1 Table, S1 Appendix), as described in the Methods section. The network was then restricted to unambiguous activating interactions only (Fig 3B), to prepare for centrality determination. Both network versions are available for download in the data repository. To analyze the resulting topology, Katz centrality was used, an algorithm suited for determining which nodes have the most incoming edges, with edges from other nodes which also have a lot of them, weighing higher than from ones which have less. Remarkably, despite the Katz centrality score being a measure of functional interaction topology only, the top results (Fig 4A) shared a high degree of similarity with top genes dictated by the miRTarBase frequency scores (Fig 4B, Table 3). The topmost result in both scoring methods was VEGFA, and they shared 5 out of their top 10 results. To establish a final ranking of genes which takes into account both miRNA targeting frequency as well as protein-protein interaction topology, the Katz centrality scores were multiplied by miRTarBase scores, giving a final combined score for the top 128 genes (Fig 4C, S2 Data). This score indicates which genes of the predicted set are expected to be most inhibited by the circulating microRNAs in ME/CFS patients (because miRNAs decrease protein abundance, the proteins which lie most downstream in an activating interaction network of the miRNA targets, will actually be the ones most inhibited by the miRNA overexpression in ME/CFS).

### Strong translational suppression of VEGF-A by circulating miRNAs implies impaired ability to adapt to hypoxia in ME/CFS patients

A sizable amount of the main findings from this analysis are consistent with experimental data in ME/CFS. Firstly, the most inhibited gene by far is VEGFA, coding for the Vascular Endothelial Growth Factor, a major secreted mediator of angiogenesis. Indeed VEGF-A levels in ME/CFS patients are decreased, as reported by Landi et al [24]. Gusnanto et al also found decreased VEGF-A as associated with ME/CFS status in a multivariate logistic regression analysis [25]. The miRNA suppression of VEGFA in ME/CFS would imply an impaired ability to adapt to hypoxia. This is of primary concern, as impaired oxygen extraction and delivery to working muscle and brain as revealed by various imaging techniques, as well as lower VO_2_ max seen in cardiopulmonary testing, are well-established findings in ME/CFS [26–33], and activity-dependent hypoxia caused by loss of functional sympatholysis has been proposed as a major mechanism involved in the disease pathophysiology [34]. A strong suppression of VEGF-A on the translational level can explain how such dysfunctions can persist chronically without angiogenic adaptation and how VEGF-A levels can not only be not elevated but actually measure lower than healthy controls who lack pathologic hypoxia. In addition to this canonical role in long-term angiogenic adaptations, recently a new function of VEGF-A has been established in the more immediate vasodilative regulation of bloodflow during muscle activity (exercise hyperemia). Skeletal muscle-specific deletion of VEGFA in mice resulted in decreased exercise capacity, without significant vascular changes, a picture reminiscent of ME/CFS [35].

Additional contribution to these dysfunctions might be the inhibition of Murine double minute 2 (MDM2), another gene scoring highly in the final ranking (Fig 4C), which is known to play a key role in the exercise-induced angiogenesis and maintaining of the capillary networks in skeletal muscle [36].

### Possible dysregulation of apoptosis and cell cycle

A large number of the genes implicated here have a role in regulating apoptosis and cell cycle. Increased apoptosis of leukocytes has been observed before in ME/CFS [37, 38], although evidence to the contrary has also been published [39], making it a contentious finding that has not been followed up in detail.The target proteins identified in the present study include both pro-apoptotic factors, such as Bim (BCL211L), c-Myc, PTEN, Foxo3 and Noxa (PMAIP1) [40–44] as well as anti-apoptotic: Bcl-2, Mcl-1, MDM2 and Akt1 [40, 45, 46]. This makes discerning directionality of the apoptosis disruption difficult, and suggests a possibility that the impact on cellular apoptosis could even be close to neutral.

As far as cell cycle dysfunctions, those have not been observed directly in ME/CFS, although they have been implicated by some genetic studies [47, 48]. The impact of circulating miRNAs on cell cycle will depend greatly on the cell types they are targeting. It is likely that they will not affect all tissues equally, especially if they are transported by exosomes, as those constitute a highly regulated method of endocrine transport, targeted to specific cells depending on the configuration of the exosomes’ tetraspanins [49]. Depending on the target tissues, the cell-cycle effects of the translational changes implicated here could either be highly significant, or very minor (if the target cells of the circulating miRNAs are fully differentiated). The lack of tissue abnormalities and structural changes in the clinical picture of ME/CFS would suggest the latter.

In total, while the involvement of apoptosis and cell cycle dysfunction may yet prove important in ME/CFS with future data, it is difficult to speculate on the specifics with currently available information.

### Circulating miRNAs as mediators of increased mitochondrial fission

What is perhaps more interesting is the growing body of new research showing that most of these same cell cycle- and apoptosis-regulating proteins play various roles in modulating cellular energy metabolism. Disruptions in mitochondrial function and cellular energy production currently constitute one of the primary research areas in ME/CFS, with many studies reporting evidence of mitochondrial dysfunction either by directly measuring oxidative phosphorylation rates [50–54], or indirectly through metabolic profiling [55–61]. Of particular interest for this study are the results presented by Schreiner et al, showing increased mitochondrial fission in cells exposed to plasma from ME/CFS patients [7]. Mitochondria are very dynamic entities, able to fragment or fuse as needed by the cell. These dynamics serve complex and diverse functions across many different cell types, though generally speaking, fused mitochondria exhibit higher energy-generating efficiency [62]. If this process is disrupted in ME/CFS cells, aberrantly shifting them more towards mitochondrial fission, this will result in lower cellular respiration capacity, (possibly explaining such observations seen in other ME/CFS studies), and causing various alterations to the normal functioning of the affected cells, which will be transiently dependent on whatever factor is causing this shift, making them difficult to study.

The results of the miRNA target analysis presented here suggest that the unknown plasma factors reported by Schreiner et al may indeed be circulating miRNAs, as many of the proteins identified in this study as likely to be inhibited the strongest by them, have effects promoting mitochondrial fusion or inhibiting fission, as follows.

Bcl-2 inhibits mitochondrial fission [63]. Mcl-1 might promote fission when overexpressed [64], however a baseline level of it is required to maintain fusion and protect from fission as well as promote normal mitochondrial respiration [65, 66]. c-Myc serves a similar role, also protecting from fission and maintaining mitochondrial oxidative phosphorylation [67]. MDM2 depletion was linked to increased mitochondrial fission through upregulated Drp1 expression [68]. The impact of Akt1 and Foxo3 on mitochondrial dynamics is unclear as they’ve been reported to promote both fusion and fission [69–71]. Noxa (PMAIP1) is the only one of the implicated apoptotic proteins which purely favors fission [72], however it is also the lowest-scoring one in this study. In addition to this, E2F1 (scoring third highest, Fig 4C), has been reported to both promote and inhibit mitochondrial fission, however the former is dependent on PINK1 [73, 74], which in turn depends on Sirt1 and can be completely suppressed by Sirt1 silencing [75, 76]. Therefore, simultaneous suppression of E2F1 and Sirt1, as implicated in this paper, will result in increased mitochondrial fission. Sirt1 itself also promotes fusion through multiple mechanisms [77, 78]. Taken all of this together, in mitochondrial dynamics a single modulatory direction of these miRNA-mediated changes can be seen much clearer than in apoptosis—suppression of c-Myc, Bcl-2, Mcl-1, MDM2, E2F1 and Sirt1 all favor increased mitochondrial fragmentation, with only the lowest-scoring Noxa pointing in the opposite direction.

### Suppression of manganese-dependent superoxide dismutase and sirtuin-1 may explain increased oxidative stress and circadian dysfunctions in ME/CFS

Another finding of same study by Schreiner et al was the reduced levels of manganese-dependent superoxide dismutase (SOD2) [7], which also corroborates the findings presented here as SOD2 is one of the highest scoring genes in the final analysis (Fig 4C), indicating a significant level of both direct and indirect suppression thereof by circulating miRNAs. Decreased SOD2 implies an impairment in antioxidant defense, especially when coupled with the suppression of Sirt1, which positively regulates many antioxidant enzymes [79, 80]. This can serve to explain the widely reported elevated markers of oxidative stress in ME/CFS [57, 81–93].

Downregulation of Sirt1 can also begin to elucidate the numerous sleep dysfunctions seen in ME/CFS patients, as it is intimately intertwined with many molecular mediators of the circadian clock [94]. Additionally, inhibition of the MAP kinase-interacting serine/threonine-protein kinase 2 (MKNK2), another gene scoring highly in the final ranking (Fig 4C) might contribute to it as well, as it is a protein known to exhibit high circadian rhythmicity [95].

### Multi-pronged suppression of TGF-β signaling

A surprising finding was the presence of TGF-β receptors in the miRNA targets list. Type II receptor (TGFBR2) had an initial score of 7 and qualified for Katz centrality analysis where it ranked highly (Fig 4C), while type I (TGFBR1) and type III (TGFBR3) receptors did not, but were still present in the initial miRTarBase results with fairly high scores of 4 and 5, respectively. As the three receptors share very little sequence similarity, this is unlikely to be a false positive. Even more so when considering that Smads, which are downstream effectors of TGF-β receptors, were also present in the results, with Smad7 scoring 5; Smad4, Smad3, Smad5 all scoring 3; Smad6 scoring 2 and Smad2 scoring 1.

TGF-β is one of the very few cytokines which has been consistently observed as elevated in ME/CFS patients [96, 97], and naturally the discussion around its role in the disease unfolded in the context of increased TGF-β signaling. The results presented here suggest that the opposite might be true, with TGF-β signaling being extensively suppressed on the translational level of all the receptor proteins as well as many of their downstream effectors, and the high levels of the cytokine itself might be compensatory. Alternatively, if the circulating miRNAs show specific affinity for only certain cell types, it might create a situation where in those targeted tissues TGF-β signaling is suppressed, while everywhere else it’s enhanced because of the high TGF-β cytokine levels.

### The shift in circulating miRNA signatures may be a result of latent herpesvirus activity

As the results presented here offer an appreciable degree of agreement with Schreiner et al, [7] the circulating miRNAs are likely to constitute, at least partially, the unknown plasma mediators implicated by their study. This offers unique insight into their possible origin, as Schreiner et al have demonstrated the same phenotype appearing when exposing cells to ME/CFS plasma as when subjecting them to culture medium from cells with transactivated HHV-6 (without carrying over any of the virus). The herpesviridae family has a known ability to alter host miRNome. This ability is not limited to activity under the canonical lytic replication cycle and can be exhibited in various non-replicative states, such as Epstein-Barr (EBV) type III latency [98] and HHV-6 transactivation [99]. If the changes in miRNA were indeed caused by latent herpesviruses, this would also line up with the ME/CFS model proposed earlier this year by Lerner and Beqaj, in which nonpermissive herpesviruses influence host cell apoptosis [100].

To explore this possibility, the previously presented miRNA-target and protein-protein interactions were used in reverse, to rank each miRNA based on their contribution to the end phenotype, as detailed in the Methods section. Higher scores were given to miRNAs contributing to the suppression of many genes with high final scores (Fig 4C), either directly or indirectly.

As revealed by a literature review, 8 of the 10 topmost miRNAs in this ranking, have been proven to be upregulated by various herpesviruses, such as the Epstein-Barr virus (EBV), human herpesvirus 6A (HHV-6A), human cytomegalovirus (CMV) and Sarcoma Kaposi’s sarcoma-associated herpesvirus (KSHV). Many were modulated by more than one virus, and some of these interactions were observed in a latent state. Although a few of the miRNAs were also downregulated at times, the clear trend is one of upregulation (Table 5). This establishes the plausibility of a herpesvirus origin of the circulating miRNA changes in ME/CFS.

**Table 5:** Existing literature on the relationship of various herpesviruses with the 10 highest-ranking circulating miRNAs.

miRNA	Ranking score	Evidence for modulation by herpesviruses.
hsa-miR-93-5p	57.45	Upregulated by EBV in gastric carcinoma [101, 102].
hsa-miR-92a-3p	46.55	Upregulated by CMV and HHV-6A in skin fibroblasts [103].
		Upregulated by EBV and KSHV in lymphomas, as well as in exosomes from them [104].
		Upregulated by latent EBV in nasopharyngeal carcinoma [105].
		Upregulated by KSHV in Kaposi’s sarcoma cells [106].
		Downregulated by latent CMV in CD34^+^ cells [107].
hsa-miR-34a-5p	35.75	Upregulated by EBV and KSHV in lymphoma cells, including by EBV type III latency [98,108].
		Upregulated by HHV-6A in T-cells [109].
hsa-miR-21-5p	34.18	Upregulated by latent EBV in lymphomas and nasopharyngeal carcinomas [98,110].
		Upregulated by KSHV latent protein K15M in human embryonic kidney cells [111].
		Upregulated by early HHV-6A infection in NK cells [112].
hsa-let-7g-5p	29.72	Upregulated by early HHV-6A and CMV infection in skin fibroblasts [103].
		Upregulated by latent EBV in nasopharyngeal carcinoma [105].
hsa-let-7d-5p	27.85	Upregulated by latent EBV in nasopharyngeal carcinoma [105].
hsa-miR-15a-5p	26.42	Upregulated by early HHV-6A infection in NK cells [112].
		Downregulated by latent EBV in NK/T-cell lymphoma [113].
hsa-miR-181b-5p	26.14	Downregulated by EBV in NK/T-cell lymphoma [114].
hsa-miR-185-5p	24.14	Possibly downregulated by EBV in megakaryocytes [115].
hsa-miR-374a-5p	23.88	Upregulated by early HHV-6A infection in skin fibroblasts [103].

Full list of scores available in S3 Data.

ME/CFS is associated with herpesviruses in the context of onset—EBV infection is the most commonly reported trigger for ME/CFS, however ongoing active infections are not often found later in the disease. Therefore, a herpesvirus origin of circulating miRNA alterations in ME/CFS would revolve around latent activity, perhaps involving unique latency conditions. A clue to this is the fact that E2F1 is strongly inhibited by the miRNAs analyzed here (Fig 4). E2F1 is usually upregulated by herpesviruses [116, 117]. HHV-6, in particular, uses it to express viral genes, making it play a crucial role in replication [118]. Therefore, the E2F1 inhibition can be a way of enforcing latency of the herpesviruses in ME/CFS. This is not an entirely new notion, the phenomenon of herpesvirus-originating non-coding RNA suppressing the replication of the same virus through various mechanisms has already been observed [119–121]. In summary, while other causes of the circulating miRNA shift in ME/CFS cannot be excluded, the connection to herpesvirus latency warrants further research. Which virus is responsible for the modulation, and from what tissue, is something that can be different from patient to patient and therefore contribute to the heterogeneity of miRNA signatures seen between patients. As herpesviruses can express their own viral miRNAs in addition to modulating the host’s, presence of those in ME/CFS ought to be investigated as well.

### High degree of cell-type dependent modulation of the miRNA-mediated changes requires further study

Amongst the most implicated genes in this study, a high amount of transcription factors can be seen, along with some epigenetic modulators. As many transcription factors exhibit complex interactions with each other, the end result is often dependent on the specific levels of each protein and presence of cell type-specific cofactors [122, 123], making the full downstream effects of the miRNA-mediated suppression of transcription factors presented here exceedingly difficult to predict computationally. They will depend greatly on the predominantly targeted cell types.

This is further complicated by the presence of the small cluster of AGO1, DICER1, DDX6 and TNRC6A in the results (Fig 2C). Those four are key proteins involved in the process of miRNA creation itself [124, 125], suggesting that the circulating miRNAs in ME/CFS might suppress other miRNAs. The full consequences of this will be dependent on the previous baseline miRNA signature of the cell and will therefore be cell-type specific as well.

The present study is also fundamentally limited by the incompleteness of miRTarBase, which received criticism for being biased towards cancer-related miRNAs, as cancer research is the main source of paired miRNA-target data. While the interactions present there are likely to be correct, many undoubtedly are missing, some of which might be of great importance to ME/CFS.

For all of the above reasons, it is recommended that further study into the effects of circulating miRNAs in ME/CFS be conducted experimentally, by exposing cultures of various cell types suspected to play a role in ME/CFS (such as skeletal muscle myocytes, neurons, microglia, macrophages, mast cells, leukocytes and various gastrointestinal cells) to the serum of ME/CFS patients and/or the exosome fraction thereof, observing the resultant changes in gene expression.

## Supporting information

S1 Data Results of per gene targeting frequency of miRNAs. (CSV)

S2 Data Full Katz centrality and combined scores. (ZIP)

S3 Data miRNA influence scores. (CSV)

S1 Table Manual literature review for protein Interactions. (CSV)

S1 Appendix References of the supporting information (mainly for S1 Table). (PDF)
