# Supplementary material for: Heterogenous circulating miRNA changes in ME/CFS converge on a unified cluster of target genes: A computational analysis
Source: PLoS One. 2023 Dec 29;18(12):e0296060. doi: 10.1371/journal.pone.0296060 (PMC10756525; doi:10.1371/journal.pone.0296060)
Supplement: S1 Appendix — (PDF) [file pone.0296060.s005.pdf]

References of the Supporting Information

1. Matsui M, Li L, Janowski BA, Corey DR. Reduced Expression of Argonaute 1, Argonaute 2 and TRBP Changes Levels and Intracellular Distribution of RNAi Factors. Sci Rep. 2015 Aug 5;5(1):12855.
2. Su F, He W, Chen C, Liu M, Liu H, Xue F, et al. The long non-coding RNA FOXD2-AS1 promotes bladder cancer progression and recurrence through a positive feedback loop with Akt and E2F1. Cell Death Dis. 2018 Feb 14;9(2):1–17.
3. Lee KH, Chen YL, Yeh SD, Hsiao M, Lin JT, Goan YG, et al. MicroRNA-330 acts as tumor suppressor and induces apoptosis of prostate cancer cells through E2F1-mediated suppression of Akt phosphorylation. Oncogene. 2009 Sep;28(38):3360–70.
4. Longo PG, Laurenti L, Gobessi S, Sica S, Leone G, Efremov DG. The Akt/Mcl-1 pathway plays a prominent role in mediating antiapoptotic signals downstream of the B-cell receptor in chronic lymphocytic leukemia B cells. Blood. 2008 Jan 15;111(2):846–55.
5. Chen G, Park D, Magis AT, Behera M, Ramalingam SS, Owonikoko TK, et al. Mcl-1 interacts with Akt to promote lung cancer progression. Cancer Res. 2019 Dec 15;79(24):6126–38.
6. Cantley LC, Neel BG. New insights into tumor suppression: PTEN suppresses tumor formation by restraining the phosphoinositide 3-kinase/AKT pathway. Proc Natl Acad Sci. 1999 Apr 13;96(8):4240–5.
7. Chin YR, Yuan X, Balk SP, Toker A. PTEN-DEFICIENT TUMORS DEPEND ON AKT2 FOR MAINTENANCE AND SURVIVAL. Cancer Discov. 2014 Aug;4(8):942–55.
8. Dobson M, Ramakrishnan G, Ma S, Kaplun L, Balan V, Fridman R, et al. Bimodal regulation of FoxO3 by AKT and 14-3-3. Biochim Biophys Acta BBA - Mol Cell Res. 2011 Aug 1;1813(8):1453–64.
9. Zemanovic S, Ivanov MV, Ivanova LV, Bhatnagar A, Michalkiewicz T, Teng RJ, et al. Dynamic Phosphorylation of the C Terminus of Hsp70 Regulates the Mitochondrial Import of SOD2 and Redox Balance. Cell Rep. 2018 Nov 27;25(9):2605-2616.e7.
10. Zhu J, Blenis J, Yuan J. Activation of PI3K/Akt and MAPK pathways regulates Myc-mediated transcription by phosphorylating and promoting the degradation of Mad1. Proc Natl Acad Sci. 2008 May 6;105(18):6584–9.
11. Saoncella S, Tassone B, Deklic E, Avolio F, Jon C, Tornillo G, et al. Nuclear Akt2 Opposes Limbal Keratinocyte Stem Cell Self-Renewal by Repressing a FOXO-mTORC1 Signaling Pathway. STEM CELLS. 2014;32(3):754–69.
12. Meulmeester E, Pereg Y, Shiloh Y, Jochemsen AG. ATM-Mediated Phosphorylations Inhibit Mdmx/Mdm2 Stabilization by HAUSP in Favor of p53 Activation. Cell Cycle. 2005 Sep 22;4(9):1166–70.
13. Xu W, Chen H, Du K, Asahara H, Tini M, Emerson BM, et al. A Transcriptional Switch Mediated by Cofactor Methylation. Science. 2001 Dec 21;294(5551):2507–11.
14. Deng M, Zeng C, Lu X, He X, Zhang R, Qiu Q, et al. miR-218 suppresses gastric cancer cell cycle progression through the CDK6/Cyclin D1/E2F1 axis in a feedback loop. Cancer Lett. 2017 Sep 10;403:175–85.

15. Ralph WM, Liu K, Auborn KJ. CCAAT/enhancer-binding protein β represses human papillomavirus 11 upstream regulatory region expression through a promoter-proximal YY1-binding site. J Gen Virol. 2006;87(1):51–9.
16. Winnik S, Gaul DS, Siciliani G, Lohmann C, Pasterk L, Calatayud N, et al. Mild endothelial dysfunction in Sirt3 knockout mice fed a high-cholesterol diet: protective role of a novel C/EBP-β-dependent feedback regulation of SOD2. Basic Res Cardiol. 2016 Apr 12;111(3):33.
17. Peng L, Yuan Z, Ling H, Fukasawa K, Robertson K, Olashaw N, et al. SIRT1 Deacetylates the DNA Methyltransferase 1 (DNMT1) Protein and Alters Its Activities ▿. Mol Cell Biol. 2011 Dec;31(23):4720–34.
18. Robertson KD, Ait-Si-Ali S, Yokochi T, Wade PA, Jones PL, Wolffe AP. DNMT1 forms a complex with Rb, E2F1 and HDAC1 and represses transcription from E2F-responsive promoters. Nat Genet. 2000 Jul;25(3):338–42.
19. Zhang Z, Wang H, Li M, Rayburn ER, Agrawal S, Zhang R. Stabilization of E2F1 protein by MDM2 through the E2F1 ubiquitination pathway. Oncogene. 2005 Nov;24(48):7238–47.
20. Tian X, Chen Y, Hu W, Wu M. E2F1 inhibits MDM2 expression in a p53-dependent manner. Cell Signal. 2011 Jan 1;23(1):193–200.
21. Leung JY, Ehmann GL, Giangrande PH, Nevins JR. A role for Myc in facilitating transcription activation by E2F1. Oncogene. 2008 Jul;27(30):4172–9.
22. Suzuki T, Kimura A, Nagai R, Horikoshi M. Regulation of interaction of the acetyltransferase region of p300 and the DNA-binding domain of Sp1 on and through DNA binding. Genes Cells. 2000;5(1):29–41.
23. Fu W, Ma Q, Chen L, Li P, Zhang M, Ramamoorthy S, et al. MDM2 Acts Downstream of p53 as an E3 Ligase to Promote FOXO Ubiquitination and Degradation. J Biol Chem. 2009 May 22;284(21):13987–4000.
24. Girnita L, Girnita A, Larsson O. Mdm2-dependent ubiquitination and degradation of the insulin-like growth factor 1 receptor. Proc Natl Acad Sci. 2003 Jul 8;100(14):8247–52.
25. Marzi I, Cipolleschi MG, D'Amico M, Stivarou T, Rovida E, Vinci MC, et al. The involvement of a Nanog, Klf4 and c-Myc transcriptional circuitry in the intertwining between neoplastic progression and reprogramming. Cell Cycle. 2013 Jan 15;12(2):353–64.
26. Cui J, Sun W, Hao X, Wei M, Su X, Zhang Y, et al. EHMT2 inhibitor BIX-01294 induces apoptosis through PMAIP1-USP9X-MCL1 axis in human bladder cancer cells. Cancer Cell Int. 2015 Feb 4;15(1):4.
27. Chang CJ, Freeman DJ, Wu H. PTEN Regulates Mdm2 Expression through the P1 Promoter *. J Biol Chem. 2004 Jul 9;279(28):29841–8.
28. Johnson-Pais T, Degnin C, Thayer MJ. pRB induces Sp1 activity by relieving inhibition mediated by MDM2. Proc Natl Acad Sci. 2001 Feb 27;98(5):2211–6.
29. Grönroos E, Terentiev AA, Punga T, Ericsson J. YY1 inhibits the activation of the p53 tumor suppressor in response to genotoxic stress. Proc Natl Acad Sci. 2004 Aug 17;101(33):12165–70.
30. Liao WR, Hsieh RH, Hsu KW, Wu MZ, Tseng MJ, Mai RT, et al. The CBF1-independent Notch1 signal pathway activates human c-myc expression partially via transcription factor YY1. Carcinogenesis. 2007 Sep 1;28(9):1867–76.
